# Extracranial Meningioma in the Scalp with Concurrent Steatocystoma

**DOI:** 10.1155/2020/6539064

**Published:** 2020-09-08

**Authors:** Jiankun Tong, Sergei A. Aksenov, Mitchell I. Chorost, William H. Rodgers

**Affiliations:** ^1^Department of Pathology, New York Presbyterian Queens, 56-45 Main Street, Flushing, NY 11355, USA; ^2^Department of Surgery, St. Francis Hospital, 100 Port Washington Blvd., Roslyn, NY 11576, USA; ^3^Weill Cornell Medical College, 525 East 68th Street, Box 130, New York, NY 10065, USA

## Abstract

This report documents a rare case of an extracranial meningioma on the posterior scalp without apparent dural connection. Additionally, a sebaceous steatocystoma of the anterior scalp presented alongside the meningioma. A steatocystoma localized to the scalp is also remarkably rare. To our knowledge, this is the first report documenting *both* an extracranial meningioma and a steatocystoma presenting concurrently on the scalp. A male patient in his thirties presented with a mass lesion on the scalp. A CT scan revealed one posterior scalp mass with no intracranial abnormalities. Post excision histologic examination confirmed an extracranial meningioma (meningothelial variant, WHO Grade I). A second anterior scalp mass, not revealed by CT scan, was discovered during surgery. It was excised and diagnosed as a steatocystoma. Meningiomas predominantly occur intracranially but, in some instances, may present as a standalone extracranial tumor *without* intracranial abnormalities. Because extracranial meningioma is uncommon, it may be overlooked during clinical diagnosis of scalp masses. We recommend that this neoplasm be routinely considered in the differential diagnosis of extracranial tumors. The discovery of another rare tumor—a steatocystoma located in immediate proximity on the scalp—is further remarkable. We briefly review relevant case reports and etiologies and consider a potential relationship between the two neoplasms. However, it remains more likely that the concurrence of these tumors in our patient was simply coincidental.

## 1. Introduction

Meningiomas are common tumors arising from meningothelial cells surrounding the brain and spinal cord. These tumors account for 24-35% of all primary brain neoplasms [1, 2]. Atypical (WHO Grade 2) and anaplastic (WHO Grade 3) meningiomas are less common, while benign (WHO Grade 1) meningiomas account for ~80% of cases [3]. While most occur in the subdural space, they can also develop extracranially without apparent dural connection [4–7]. In such cases, they are called extracranial or ectopic meningiomas. Histologically, intracranial and extracranial meningiomas are indistinguishable [8]. Primary extracranial meningiomas are rare, slow-growing tumors that account for fewer than 2% of all meningiomas [3, 9]. Etiology is debatable, but several pathways are proposed, including migration of meningothelial cells beyond the skull and meningothelial differentiation of pluripotent mesenchymal cells [3]. Complete cure can be attained by surgical excision, and prognosis is generally excellent [3, 10]. Accumulating case reports of extracranial meningiomas [3–13] call for routine consideration of this neoplasm in differential diagnoses of soft tissue tumors on the scalp.

Steatocystoma is an uncommon cutaneous disorder presenting as single or multiple sebaceous duct cysts. Typically, these cysts appear in anatomical areas rich in sebaceous glands [14]. Interestingly, whilst the scalp is rich in sebaceous glands, instances of steatocystomas localized to the scalp are extremely rare. Prior to 2013, only 12 cases were documented [15], though more cases were recently published (e.g., [14, 16]). Steatocystoma is benign and can be left untreated; however, it can present a significant cosmetic distress [15, 16].

In this report, we describe a case of a large extracranial meningioma presented as a posterior scalp mass coexisting with a solitary steatocystoma of the anterior scalp. The latter was discovered unintentionally during surgery. Both conditions are rather rare, and to the best of our knowledge, this is the first report documenting an extracranial meningioma concurrent with a steatocystoma on the scalp.

## 2. Case Presentation

A male patient in his thirties presented with a mass lesion on the scalp. This was a recurrent mass lesion on the posterior scalp (~3–4 cm at the greatest dimension), status post incision and drainage in the remote past (>10 years ago). Currently, the patient sought treatment as the lesion seemed to increase in size and was associated with pain. Past medical history as well as family history was unremarkable.

**Figure 1:**
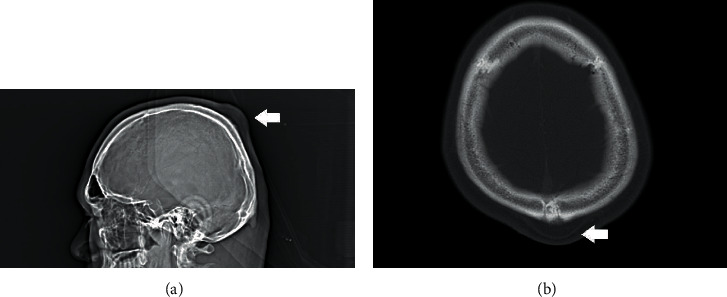
Computed tomography of the cranium showing a posterior scalp mass at the midline near the convexity: (a) sagittal view; (b) transverse view: 3.7 cm at the greatest dimension.

A CT scan of the brain with contrast revealed a posterior scalp mass at the midline near the convexity, which was inseparable from the calvarium (3.7 cm at the greatest dimension; Figure 1). The underlying calvarium was unremarkable. There was no evidence of intracranial mass effect or increased intracranial pressure. Regional lymph nodes of the head and neck were also unremarkable (i.e., not enlarged). Overall, the radiologic impression was posterior subgaleal lipoma. The differential diagnosis also included benign adnexal tumor, malignant melanoma, and benign smooth muscle tumor. The posterior mass lesion was firmly attached to the periosteum of the scalp, and complete excision and hemostasis were achieved using Bovie cautery.

**Figure 2:**
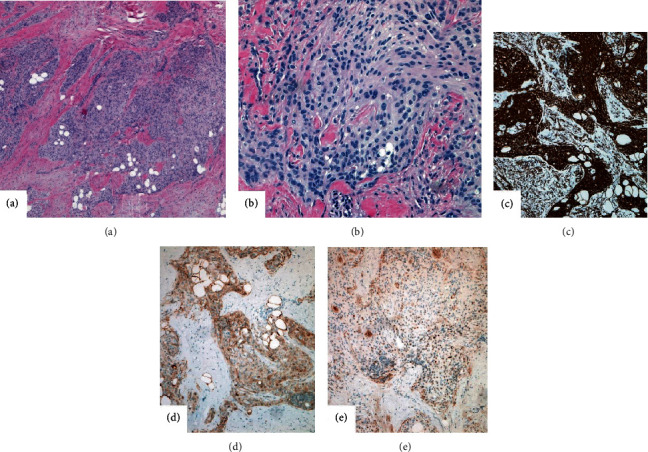
(a) Cords and nests of tumor cells are surrounded by dense fibrotic stroma and adipose tissue; hematoxylin-eosin, original magnification ×40. (b) The meningothelial cells are spindly looking with pink cytoplasm. Cells are packed together forming syncytial structures and whorls; hematoxylin-eosin, original magnification ×200. (c) Tumor cells show strong and diffuse cytoplasmic staining with vimentin (magnification ×100), (d) weak to moderate cytoplasmic and nuclear staining with S-100 (magnification ×100), and (e) focal weak to moderate nuclear staining with progesterone receptor (PR) (magnification ×100).

Histologic examination of the posterior scalp mass showed cells forming syncytial structures and whorling growth patterns in the subcutaneous adipose tissue. Cells were spindly looking with moderate amount of amphophilic cytoplasm, arranged in cords and nests, separated by a thick collagenous stroma and adipose tissue (Figure 2(a)). The nuclei showed relatively bland features with fine chromatin and indistinct nucleoli (Figure 2(b)). Rare psammomatous calcifications were also noted. No mitotic figures were seen. Tumor cells were immunohistochemically strongly positive for vimentin (Figure 2(c)) and weakly positive for S-100 (Figure 2(d)) and progesterone receptor (PR) (Figure 2(e)), while negative for AE1/3, CAM 5.2, EMA, HMB-45, Melan-A, and SMA. The Ki67 proliferative index was <5%. The final pathologic diagnosis on the posterior scalp mass was extracranial meningioma, meningothelial variant, WHO Grade 1.

**Figure 3:**
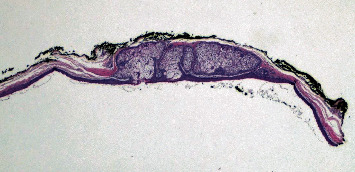
Sebaceous glands contiguous with the cyst wall. Hematoxylin-eosin, original magnification ×40.

During surgery, a second lesion was found at the anterior scalp. This finding was incidental, as no radiographic abnormalities at the anterior scalp were described. This was a smaller subcutaneous lesion approximately 1 cm at the greatest dimension. It was removed at time of surgery without technical difficulties. Histologically, this anterior scalp mass showed a cystic lesion with sebaceous glands adjacent to the cyst wall (Figure 3). It was diagnosed as a steatocystoma.

The patient received local radiation therapy as resection margins were focally positive for tumor. The 5-year follow-up showed no recurrence of meningioma or steatocystoma, and the patient did not present further complications.

## 3. Discussion

Meningioma is the most common and well-known intracranial tumor in the central nervous system [1]. It can also occur extracranially, presenting as a soft tissue nodule or mass (extracranial meningioma, cutaneous meningioma, scalp meningioma, or ectopic meningioma) [3–7, 9, 11–13]. Extracranial variants, however, only occur in fewer than 2% of all meningioma cases [3, 9]. Therefore, this neoplasm can be easily overlooked during the differential diagnosis of scalp masses.

Although the patient had prior incision and drainage on the posterior mass in the past, no tissue was submitted for pathologic examination at that time. Imaging studies demonstrated no evidence of intracranial abnormalities, and the radiographic impression suggested subgaleal lipoma. Subgaleal lipoma is a heterotopic tumor of adipose tissue appearing between the galea and periosteum of the frontal bone. It is usually painless and not adjacent to the calvarium. Our patient, however, presented a painful, posteriorly located lesion, adjacent to the calvarium. Hence, we assumed it may be representative of an extracranial meningioma with cystic change or another cystic lesion. Ultimately, complete excision of the mass/lesion with pathologic examination (including immunohistochemistry) is essential in confirming a definite diagnosis. Hematoxylin and eosin sections suggested a low-grade meningioma (see Figures 2(a) and 2(b)). To confirm this interpretation (particularly due to the unusual location of the tumor), a standard immunohistochemistry panel was used. Indeed, extracranial meningioma was confirmed (see Figures 2(c)–2(e)).

Extracranial meningiomas can be associated with an intracranial mass or other intracranial anomalies. Our patient, however, showed no intracranial abnormalities. Indeed, on the scalp, extracranial meningioma can also present as a standalone tumor [4–7, 10–13]. Thusly, lack of a concurrent intracranial mass or evidence of intracranial anomalies should not exclude extracranial meningioma, as depicted in our case. We strongly urge that this neoplasm be routinely considered in the differential diagnosis of soft tissue scalp masses.

A second mass lesion, not revealed by radiographic examination, was discovered on the anterior scalp during surgery. Pathologic examination concluded a steatocystoma. Steatocystoma (or sebaceous duct cyst) is an uncommon benign tumor. The typical form is hereditary and linked to a mutation in the coding region of the *KR17* gene [14]. It appears primarily in adolescence [15, 17] and can affect the trunk, head, neck, and earlobes [14, 18]. Notably, in our case, steatocystoma was found on the anterior scalp at an adult age. Cases of steatocystoma limited to the scalp are sporadic (i.e., nonhereditary) and appear in adulthood, such as in our patient. A steatocystoma limited to the scalp is exceptionally rare [14–17], and a concurrent finding of this tumor alongside an extracranial meningioma—another rare tumor—is further remarkable.

One case report described an adult male patient with a recent history of *intracranial* (parasagittal) meningioma, presenting with steatocystoma on the scalp and facial area [14]. The possibility of interrelation between the two neoplasms was not explored at the time [14]. Indeed, there are no conclusive reports concerning etiology or comorbidity of nonhereditary steatocystoma [17]. However, one group suggested that immunological events might be among the causative factors [19]. Interestingly, studies show that meningiomas often contain substantial immune cell infiltrates [20] including T cells, B cells, plasma cells, and macrophages [20–22]. Thusly, it is not implausible to suggest that immunological events in the tumor microenvironment of a meningioma might potentially contribute to the onset of steatocystoma in proximal tissues (e.g., the scalp). Naturally, barring strong clinical and/or experimental evidence, this mechanism, albeit tenable, remains entirely speculative.

The sheer lack of cases reporting concurrence of extracranial meningioma and steatocystoma precludes any further meta-analysis at present. Given that causative factors and etiologies of both neoplasms remain largely unclear, and no conclusive comorbidity mechanisms can be established, it is likely that the concurrence of these conditions in our patient was simply coincidental. Yet, we find value in publishing our findings, as similar cases in the future might benefit from this report.

## 4. Conclusion

1. In our case, the clinical differential diagnosis included several mesenchymal tumors such as lipoma, benign adnexal tumor, malignant melanoma, and benign smooth muscle tumor. Extracranial meningioma was not considered. Awareness that meningiomas may occur in an extracranial location without concurrent intracranial abnormalities will help avoid potential mismanagement of these neoplasms due to being overlooked. Extracranial meningioma appears indistinguishable from other tumors based on radiological examination. While relatively rare, it is nonetheless essential that clinical teams always consider extracranial meningioma in the differential diagnosis of scalp masses
2. We found no prior case reports documenting a steatocystoma on the scalp concurrent with an extracranial meningioma. Both tumors are rare, and to the best of our knowledge, this is the first case report describing the coexistence of these neoplasms alongside each other on the scalp. We speculated on the possibility that immunological events often associated with meningiomas might play a potential causative role in the onset of steatocystoma in the immediate anatomical proximity. Due to the scarcity of similar case reports and lack of additional relevant evidence, the suggested mechanism is weakly supported and entirely speculative. However, we hope that future studies might benefit from this publication

## Data Availability

The authors of this manuscript confirm that the data supporting the conclusions of this study are found within the article and by consulting the works cited.
